# Change in sensory integration and regularity of postural sway with the suspensory strategy during static standing balance

**DOI:** 10.3389/fneur.2023.1290986

**Published:** 2023-10-31

**Authors:** Linjing Jiang, Satoshi Kasahara, Tomoya Ishida, Yuta Koshino, Ami Chiba, Kazumasa Yoshimi, Yuting Wei, Mina Samukawa, Harukazu Tohyama

**Affiliations:** ^1^Department of Rehabilitation Sciences, Graduate School of Health Sciences, Hokkaido University, Sapporo, Japan; ^2^Department of Rehabilitation Sciences, Faculty of Health Sciences, Hokkaido University, Sapporo, Japan; ^3^Department of Rehabilitation, Hirosaki University Hospital, Hirosaki, Japan

**Keywords:** sensory integration, sensory input, sensory weights, time-frequency analysis, postural regularity, sample entropy, suspensory strategy, knee flexion

## Abstract

**Background and aim:**

The suspensory strategy, a method for controlling postural balance in the vertical direction of the center of mass (COM), is considered by the elderly as a means of balance control. The vertical COM control might alter the sensory integration and regularity of postural sway, which in turn impacts balance. However, to date, this was not confirmed. Thus, this study aimed at investigating the influence of the suspensory strategy achieved through knee flexion on the static standing balance.

**Methods:**

Nineteen participants were monitored at knee flexion angles of 0°, 15°, and 65°. Time-frequency analysis and sample entropy were employed to analyze the COM data. Time-frequency analysis was utilized to assess the energy content across various frequency bands and corresponding percentage of energy within each frequency band. The outcomes of time-frequency are hypothesized to reflect the balance-related sensory input and sensory weights. Sample entropy was applied to evaluate the regularity of the COM displacement patterns.

**Results:**

Knee flexion led to a decreased COM height. The highest energy content was observed at 65° knee flexion, in contrast with the lowest energy observed at 0° in both the anterior–posterior (AP) and medial-lateral (ML) directions. Additionally, the ultra-low-frequency band was more pronounced at 65° than that at 0° or 15° in the ML direction. Furthermore, the COM amplitudes were notably higher at 65° than those at 0° and 15° in the AP and ML directions, respectively. The sample entropy values were lower at 65° and 15° than those at 0° in the ML direction, with the lowest value observed at 65° in the vertical direction.

**Conclusion:**

The suspensory strategy could enhance the sensory input and cause sensory reweighting, culminating in a more regular balance control. Such suspensory strategy-induced postural control modifications may potentially provide balance benefits for people with declining balance-related sensory, central processing, and musculoskeletal system functions.

## 1. Introduction

Maintaining upright balance depends on the capacity to maintain the center of pressure (COP) and/or center of mass (COM) within or anticipated to stay within the base of support (BOS) (1, 2). In this context, ankle and hip strategies established in the postural control during the standing posture effectively drive the COP and COM within the BOS, particularly in the anterior–posterior (AP) and medial-lateral (ML) directions (3). However, the COM control during fall prevention is not limited to the two-dimensional control on the horizontal plane but also involves COM vertical control (3, 4). In addition to the ankle and hip strategies, the suspensory strategy is the third postural strategy which employs flexing of the knee joints to lower the COM toward the base of support and achieve COM vertical control (3, 4). However, this suspensory strategy contributes to balance control by lowering COM, which remains a controversial topic (4–6).

The rationale of the suspensory strategy is that a flexed knee combined with flexion of the ankle and hip joints can lower the COM height and absorb internal and external perturbations; thus, effectively increasing stability and reducing the likelihood of falling (3–5). Previous studies also suggested that elderly people might be more inclined to use the suspensory strategy, such as bending the knees or lowering the COM to increase posterior stability (3, 4). However, a study has reported that different depths of squatting (i.e., different knee flexion angles) decrease the functional stability limits by inducing changes in joint mobility and muscle activation patterns (6). These divergent findings may be partially attributed to variations in the employed resources, such as COM, joint angle, and muscle activation, and assessment method. For instance, a change in COP or COM sway indicates balance ability in most studies (1, 2, 7). However, the appellate approach tends to ignore the sensory inputs and central processes in postural control. Moreover, the COP or COM sway has recently been utilized to evaluate the engagement of sensory integration, such as vision, vestibular, and proprioception, and estimate the regularity of posture sway (8–11). This represents an assessment of the potential factors that influence the postural balance. Therefore, by building upon the analysis of amplitude magnitude and integrating the sensory system and regularity of postural sway, a suspensory strategy examination could potentially offer further insights into its role in postural balance within the existing framework of postural control strategy.

Time-frequency analysis and nonlinear analysis of COP or COM data have been demonstrated as crucial tools for evaluating the overall posture system to estimate the sensory component and regularity of postural sway (10–14). Time-frequency analysis of COP and COM is recommended for studying human posture control, as it is believed to offer a better understanding of the underlying mechanisms, such as sensory input or sensory weighting in the static balance, than the traditional measures, such as amplitude, average velocity, or area (10, 13, 15). By calculating the local energy content within different frequency bands using time-frequency analysis, the sensory weightings of various sensory inputs can be assessed, and the overall energy content in the range of 0–6.25 Hz can reflect the collective balance-related sensory input, providing balance-related sensory integration insights during static standing (10, 15, 16). Sample entropy, which is a widely-used nonlinear evaluation method, has been employed for the assessment of postural regularity (14, 17, 18). It provides valuable means of quantifying the regularity and variability present in postural control, which is crucial for understanding the underlying neuromuscular mechanisms governing balance (14). Prior studies have suggested that the increase in the sample entropy of postural sway could be explained by the low level of postural sway regularity and having less attention dedicated to balance control (11, 18, 19). In conjunction with the notion that healthier balance control involves reduced attentional engagement in postural sway, which in turn leads to a greater degree of central control becoming automated (20), an elevation in postural sway entropy typically signifies enhanced balance control.

In addition, a review has reported that the COM vertical control is crucial for the standing balance and requires integration of multisensory cues that are critical for balance in vertical direct estimation, vertical perception, and stability (21). However, to date, no studies have directly addressed the sensory integration and regularity of suspensory strategy for static standing. Therefore, it is important to clarify the differences in the vertical postural control, such as the suspensory strategy, with sensory integration and regularity of postural sway from other postural controls, such as the hip and ankle postural control, for planning rehabilitation of older adults and patients with motor disorders and the design of exoskeletons considering the sensory integration and motor control of the user. Previous studies have reported that knee flexion modifies the passive stabilization achieved through ligaments and bone shape during standing and replaces it with active stabilization from muscle engagement (22). Thus, we hypothesized that suspensory strategy with knee flexion could change the sensory integration which in turn could lead to an increase in attention demand owing to the active involvement of the muscles. Accordingly, this study aimed at exploring the effect of knee joint flexion as a suspensory strategy to maintain static standing balance.

## 2. Methods

### 2.1. Participants and equipment

Based on the preliminary data, most of the parameters showed large effect sizes. Thus, the G*Power (3.1.9.7) was used with an F test. The sample size calculation revealed that a minimum of 12 samples were necessary for the experiment, with an effect size of *f* = 0.4, alpha = 0.05, and power = 0.8 (23). Nineteen healthy young adults (11 males and 8 females; mean age ± SD, 24.3 ± 2.2 years; age range, 21–30 years; weight, 62.7 ± 13.8 kg; and height, 169.5 ± 5.9 cm) were enrolled in this study. The participants with any current or past orthopedic or neurological illnesses were excluded from the study. Prior to inclusion, the participants were informed about the study and they signed a consent form for participation in this study. Consequently, all the participants were enrolled in this study. This study was approved by the review board of our institution (22–66) and was conducted in accordance with the ethical guidelines set forth by the 1964 Declaration of Helsinki.

Three-dimensional motion capture kinematic data were acquired using seven high-speed cameras (Hawk cameras, Motion Analysis Corp., Santa Rosa, CA, United States). The collected data were then analyzed using a motion analysis system (Cortex, version 5.0.1, Motion Analysis Corp.). Thirty-five retroreflective markers were placed at the specific anatomical points (24, 25). Markers were placed on the front and back of the head, shoulders, elbows, and wrists. Markers were also placed on the seven cervical vertebrae, sternum, scapula, anterior and posterior iliac spines (ASIS and PSIS, respectively), lateral thigh, medial and lateral femoral epicondyle, lateral shank, medial and lateral malleolus, second metatarsal head, fifth metatarsal head, and heel. The marker coordinate data were collected at a sampling rate of 200 Hz.

### 2.2. Procedures

**Figure 1:**
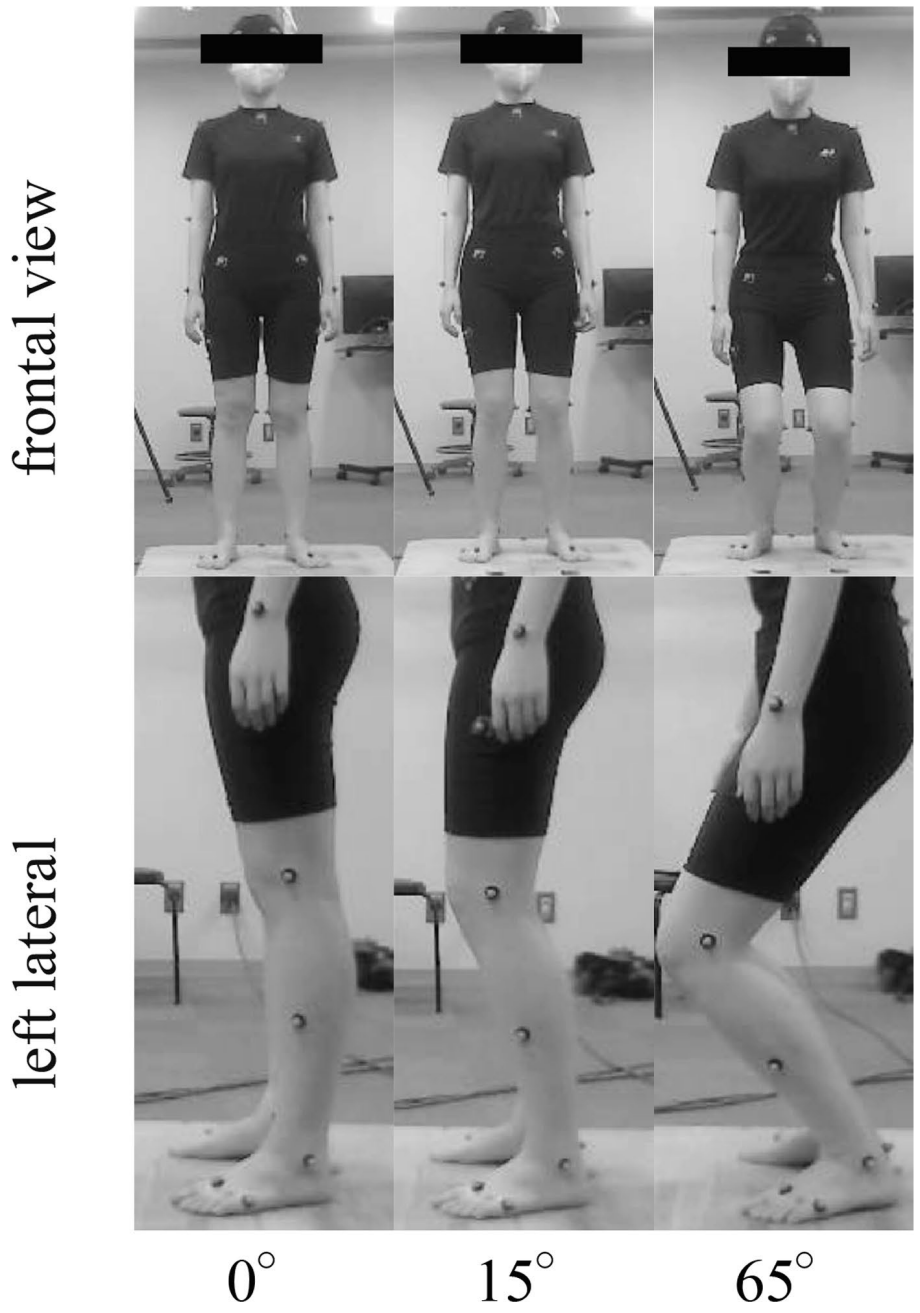
Representative pictures of a participant standing at three knee flexion angles (0°, 15°, and 65°).

To manipulate the suspensory strategy, participants were instructed to adopt three different postures with 0° (knee is locked by screw home mechanism) (22, 26), 15° (provide active stabilization and enable elastic absorption) (5, 22), and 65° (with lower stiffness, which may lead to separation of the head from the whole-body movement) (27) of knee flexion (Figure 1). The feet were positioned at the same place with the interval as the hip width, and had the same orientation in all postures. No specific instructions were provided regarding the other joints. No specific instructions were provided regarding the other joints. To stabilize their gaze and avoid exceeding the bending posture, participants were asked to gaze at the fixation point with a 9-cm diameter circle at approximately 5 m in front of them across all tasks. The participants practiced the postures beforehand and received feedback from examiners to correct any errors during the trials. Knee joint angles and COM heights were verified to ensure compliance with the experimental design every trial. The experimental procedure involved asking the participants to stand in each of the three positions for at least 35 s, with the order of the positions randomized. The participants were instructed to maintain each posture with different knee flexion angles for at least 35 s, and the order of the postures was randomized. After recording the data in one posture, the participants returned to a relaxed standing posture and rested for 10 s until the next posture. Ideally, there were no rest periods between each two postures, but if the participants experienced fatigue during the experiment, a 5-min break was provided.

### 2.3. Data collection and processing

Kinematic data, including knee joint angles and COM position, were analyzed using Visual3D software (version 6, C-Motion, Inc., Germantown, MD, United States) and processed with MATLAB (MATLAB 2022b, The MathWorks, Inc., Natick, MA, United States). A fourth-order, zero-lag Butterworth filter with a 10-Hz cutoff frequency was applied to the kinematic data to obtain a low-pass filtered signal (4). For the next data processing session, 30 s of kinematic data were extracted from the recorded data 35 s after visually confirming the knee angle and postural stability. In concrete terms, the knee angle was measured using a goniometer before recording the data, and then the knee angle was confirmed using Visual3D software offline data processing after recording. If the knee angle was incorrect, participants were asked to hold their posture again. The COM sway amplitudes in the AP, ML, and vertical directions were calculated at the selected analysis intervals and normalized according to the height of each participant. The COM height was calculated as the average COM height at the selected analysis intervals and normalized to the height of each participant (4).

In accordance with prior studies (10, 13), a time-frequency analysis was conducted on the AP and ML directions of the COM to explore the sensory energy input in the current study. Specifically, the COM signal was processed using Gabor transform, which is a time-frequency analysis technique used to extract the energy content during different frequency band of the signals. The Gabor transform is shown in Eq. (1).


(1) $$G\left(t,f\right)=\overset{+\infty }{\underset{-\infty }{\int }}x\left(\tau \right)e^{-\sigma \pi {\left(t-\tau \right)}^{2}}e^{-j2\pi f\tau }d\tau$$

The Gaussian window with a width controlled by parameter σ in Equation (1) is represented by output G(t, f), which displays the central oscillator location at various frequencies. The integral of G(t, f) along the frequency axis represents the accumulation of information from different frequency bands.

The total energy content was determined by adding the energy in the frequency range of 0–6.25 Hz (10). To assess alterations in the proportion of energy content from different sensory systems, the frequency range was divided into four bands: (1) ultra-low-frequency band (below 0.1 Hz); (2) very-low-frequency band (0.10–0.39 Hz); (3) low-frequency band (0.39–1.56 Hz), and (4) moderate-frequency band (1.56–6.25 Hz), which correspond to the visual system, vestibular system, cerebellar system, and proprioception, respectively (10, 15, 27–30). The local energy content of each frequency band was calculated as a percentage of the total energy by referring to the sensory weights (10, 31, 32). Sample entropy was previously used to analyze the regularity of postural sway data during static standing (11, 33). Thus, it was calculated for the AP, ML, and vertical COM displacements. The computation method for sample entropy was implemented using an approach developed by Richman and Moorman, which was translated into MATLAB functions and utilized for further calculations (33–35). The sample entropy is shown in Equations (2–4).

(2) $$\text{Sample entropy (m,r,N) = −ln}(\frac{A}{B})$$

(3) $$\text{A = (}\frac{\left(N-m-1\right)\left(N-m\right)}{2})A^{m}\left(r\right)$$

(4) $$\text{B = }(\frac{\left(N-m-1\right)\left(N-m\right)}{2})B^{m}\left(r\right)$$

The length of the sequences to be compared is denoted by m, the tolerance value for accepting matches is denoted by r, the length of the data is denoted by N, the probability that sequences match for m + 1 point is denoted by A^m^(r), and the probability that sequences match for m point is denoted by B^m^(r). However, there is no consensus on the parameter selection, but for balance control studies, the parameter settings are commonly set to m = 2 or 3 and r between 0.1 and 0.25 standard deviation (SD) (33). For this study, the parameters were set as m = 2 and r = 0.2 multiplied by the SD (33–35). Generally, the sample entropy value approaches zero for a perfect regularity pattern. Conversely, if the signal has more irregularity, the sample entropy value tends to be higher (35, 36).

### 2.4. Statistical analyses

All statistical analyses were performed using SPSS Statistics (version 18.0; IBM Corp., Armonk, NY, United States). The Shapiro–Wilk test was conducted to test normality. One-way repeated-measures analysis of variance (ANOVA) or Friedman’s test was used to examine the effects of posture (three different degrees of knee flexion) on the outcome measure. *Post hoc* analyses between the two postures with different knee flexions were conducted using the Bonferroni paired comparison method or pairwise comparisons. For one-way repeated-measures ANOVA, Cohen suggested that small, medium, and large effects would be reflected in the partial eta squared values (η^2^) to 0.01, 0.06, and 0.14 (23). According to Cohen’s guidelines, Kendall’s W value was used for Friedman’s test, with values in the range of 0.1–0.3 indicating a small effect, values in the range of 0.3–0.5 indicating a moderate effect, and values ≥ 0.5 indicating a large effect (23). The significance level was set at a *p*-value < 0.05.

## 3. Results

Friedman’s test indicated a significant effect of the knee flexion angle on the COM height [χ^2^(2) = 38.00, *p* < 0.001, Kendall’s W = 1.00]. *Post hoc* analyses revealed a significant difference in the COM height among the three knee flexion angles (all *p* < 0.001). Specifically, the median (Q1–Q3) of the COM height were 51.62% (50.47–52.68), 50.65% (49.33–51.35), and 41.47% (39.03–43.22) at 0°, 15°, and 65° knee flexion, respectively.

Friedman’s test revealed significant differences in the AP [χ^2^(2) = 8.84, *p* < 0.05, Kendall’s W = 0.23], ML [χ^2^ (2) = 17.16, *p* < 0.001, Kendall’s W = 0.45], and vertical [χ^2^(2) = 34.11, *p* < 0.001, Kendall’s W = 0.90] direction in the COM sway among the three knee flexion angles. *Post hoc* analyses were performed to further investigate specific pairwise differences. COM sway amplitude at 0° knee flexion in the AP direction was significantly smaller than that at 65° (*p* < 0.05), and the sway amplitude at the 0° knee flexion in the ML direction angle was significantly smaller than that at both 15° and 65° (both *p* < 0.05; Table 1). The COM sway amplitude at 65° knee flexion in the vertical direction exhibited the largest amplitude, whereas the COM sway amplitude at the knee flexion angle 0° had the smallest amplitude (both *p* < 0.05; Table 1).

**Table 1:** The median (Q1, Q3) deviation for each COM amplitude and sample entropy of the AP and ML directions and the mean (standard deviation) for sample entropy vertical direction.

		Knee flexion angle		
	COM direction	0°	15°	65°
COM sway amplitude (%)	AP	1.09 (0.70, 1.31)	1.20 (0.84, 1.52)	1.61 (1.24, 2.41)^*^
	ML	0.48 (0.35, 0.82)	0.80 (0.61, 1.02)^*^	0.97 (0.82, 1.12)^*^
	Vertical	0.15 (0.10, 0.24)	0.34 (0.17, 0.44)^*^	1.06 (0.75, 1.25)^*#^
Sample entropy	AP	0.33 (0.27, 0.43)	0.35 (0.32, 0.41)	0.31 (0.26, 0.39)
	ML	0.41 (0.37, 0.53)	0.34 (0.30, 0.35)^*^	0.26 (0.21, 0.33)^*^
	Vertical	0.55 (0.20)	0.55 (0.20)	0.29 (0.09)^*#^

COM AP, COM anterior–posterior direction; COM ML, COM medial-lateral direction; Q1, first quartile; Q3, third quartile. ^*^P < 0.05, compared with 0° knee flexion; ^#^*p* < 0.05, compared with 15° knee flexion.

**Figure 2:**
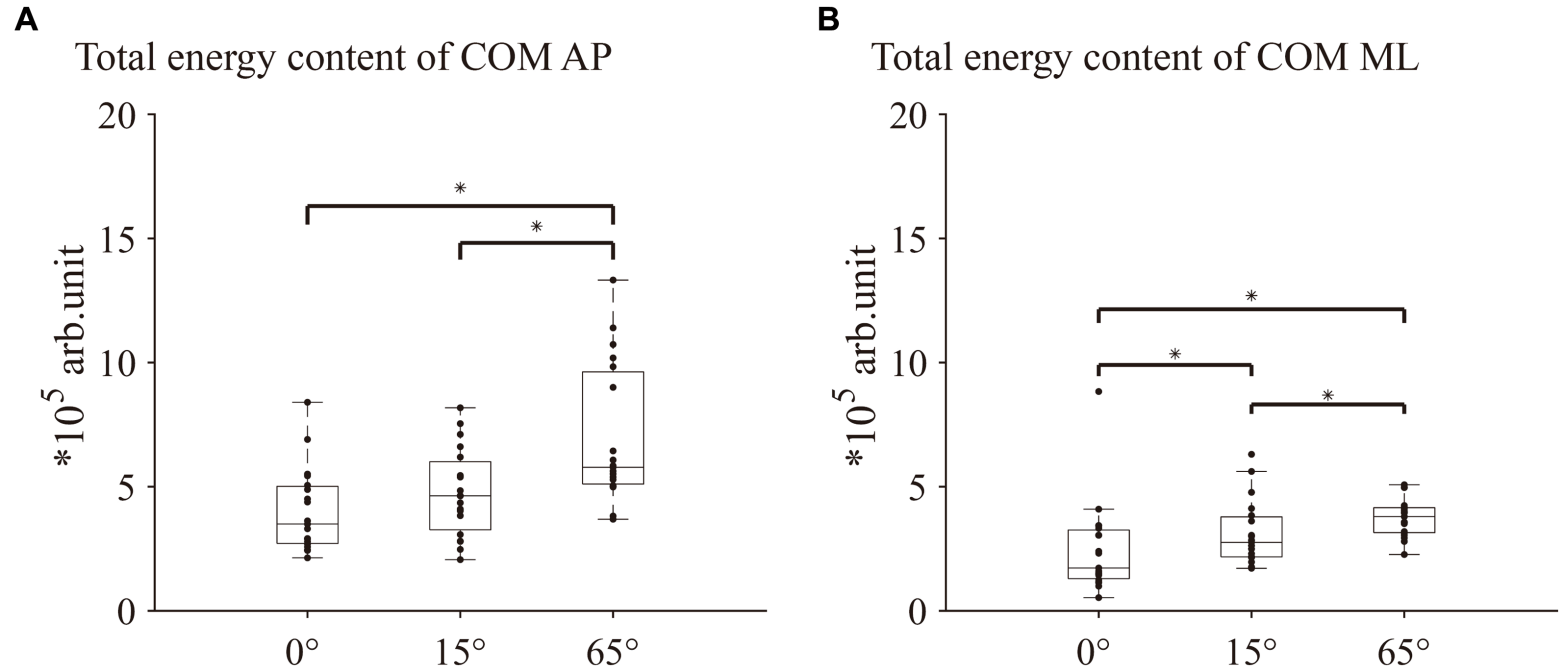
Comparison of the total energy content of COM AP and ML between 0°, 15°, and 65° knee flexion. **(A)** Total energy content of COM AP. **(B)** Total energy content of COM ML. COM AP, COM anterior–posterior direction; COM ML, COM medial-lateral direction. * indicates *p* < 0.05.

**Figure 3:**
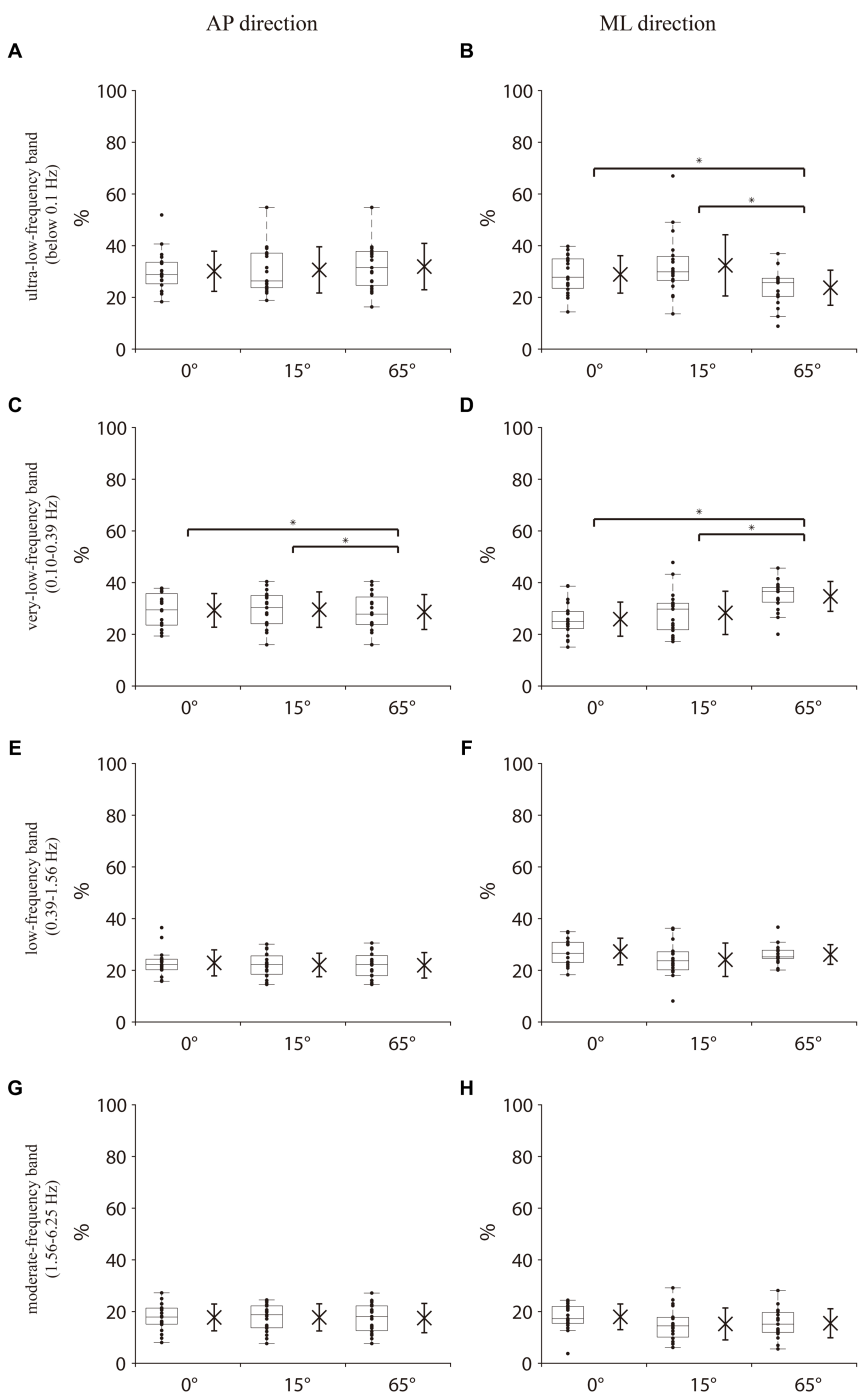
Percentage of energy content in each frequency band in the COM AP and COM ML directions. **(A,B)** Ultralow-frequency band (<0.1 Hz, visual system). **(C,D)** Very low-frequency band (0.1–0.39 Hz, vestibular system). **(E,F)** Low-frequency band (0.39–1.56 Hz, cerebellar system). **(G,H)** Moderate-frequency band (1.56–6.25 Hz, proprioception and spinal reflexive loop). The box plot represents the median values and interquartile range of the entire sample for each measurement and displays the maximum and minimum values through the upper and lower whiskers. Data for each participant are represented by dots. The x on the left side of the box plot indicates the mean of all the data and the whiskers on x indicate the standard deviation. COM AP, COM anterior–posterior direction; COM ML, COM medial-lateral direction. * indicates *p* < 0.05.

Significant differences in the total energy content of all frequency bands in the COM AP direction among the three different angles of knee flexion [χ^2^(2) = 14.632, *p* < 0.001, Kendall’s W = 0.39] were revealed. *Post hoc* analyses indicated that the total energy content at 65° knee flexion was significantly lower than that at 0° and 15° (both *p* < 0.05; Figure 2A). For the percentage of energy content of each frequency band in the COM AP direction, Friedman’s test revealed no significant differences among the three knee flexion angles in the ultralow-, low-, and moderate-frequency bands (Figures 3A,E,G). One-way repeated-measures ANOVA revealed a significant difference in the percentage of the very-low-frequency band among the three knee flexion angles [*F*(2, 36) = 5.71, *p* < 0.05, η^2^ = 0.24]. *Post hoc* analysis showed that the energy percentage of the very-low-frequency band was significantly lower at the 65° knee flexion angle than those at 0° and 15° knee flexion angles (all *p* < 0.05; Figure 3C).

Friedman’s test revealed a significant difference in the total energy content in the COM ML direction among the three knee flexion angles [χ^2^(2) = 20.632, *p* < 0.001, Kendall’s W = 0.543]. *Post hoc* analyses indicated that the total energy content at the 65° knee flexion was significantly lower than those at 0° and 15°(both *p* < 0.05; Figure 2B). For the percentage of energy content of each frequency band in the COM ML direction, no significant differences were observed in the low-and moderate-frequency bands among the three knee flexion angles (Figures 3F,H). Friedman’s test and one-way repeated measures ANOVA revealed a significant difference in the ultra-low-frequency band [χ^2^(2) = 9.58, *p* < 0.05, Kendall’s W = 0.25] and very-low-frequency band [*F*(2, 36) = 13.36, *p* < 0.001, η^2^ = 0.43] among the three knee flexion angles. *Post hoc* analysis showed that the energy percentage of the ultra-low-frequency band was significantly lower at a 65° knee flexion angle than those at 0° and 15° (both *p* < 0.05; Figure 3B), and the energy percentage of the very-low-frequency band was significantly lower at the 0° and 15° knee flexion angles than that at 65°(both *p* < 0.05; Figure 3D).

Friedman’s test was conducted to compare the sample entropy of the COM among the three knee flexion angles. There was no significant difference in the sample entropy of the COM AP direction among the three knee flexion angles [χ^2^(2) = 4.11, *p* = 0.13, Kendall’s W = 0.23]. A significant difference was observed in the COM ML direction [χ^2^(2) = 15.47, *p* < 0.001, Kendall’s W = 0.45], with the 0° knee flexion angle exhibiting a significantly higher sample entropy than those at 15° and 65° (both *p* < 0.05; Table 1). Furthermore, a significant difference was observed in the sample entropy in the vertical direction [χ^2^(2) = 21.72, *p* < 0.001, Kendall’s W = 0.87]. *Post hoc* analysis revealed that the 65° knee flexion angle displayed a significantly lower sample entropy than those at 0° and 15° (both *p* < 0.001; Table 1).

## 4. Discussion

This study analyzed COM sway using classical parameters (amplitude), time-frequency analysis, and non-linear analysis to investigate the effect of the suspensory strategy on the static standing balance by knee flexion. In the classical parameter analysis, the COM sway amplitude in the AP, ML, and vertical directions showed an increase during knee flexion. In terms of the time-frequency analysis, the results showed that the full-band energy content increased and the sensory weights changed while the knee was flexed. Thus, this work demonstrated that knee flexion alters the postural control in the vertical direction and this suspensory strategy alters the balance-related sensory integration and postural sway regularity.

### 4.1. Postural sway amplitude

During static standing, the COM sway amplitude is typically inversely related to the balance ability; thus, smaller amplitudes indicate better balance (12, 37). However, some studies have shown that postural sway amplitude can decrease during heightened tension (17), postural stiffness (37), or less attention (8), which does not necessarily indicate an improvement in the posture. Our study revealed that the COM sway amplitude in the AP and ML directions was slightly increased, and this level of increase did not seem to threaten the balance. However, there were noticeable alterations in the vertical direction by knee flexion with a substantial magnitude of change. Based on previous studies (8, 17, 37) suggesting that potential sway serves as an exploratory mechanism which ensures that multiple sensory systems provide continuous dynamic input (16, 38, 39), the small increase in COM sway amplitude in the horizontal plane in this study may imply functionality rather than improvement in or worsening of balance. The change in COM sway amplitude in the vertical direction may occur in the knee flexion state with the increased freedom of the lower limb (e.g., more freedom of movement in the knee joint in a knee flexion stance relative to a straight knee stance); thus, allowing for balance control to potentially employ more control strategies in the vertical direction of the COM, also known as suspensory strategies (3, 4). However, there is a TRADE-OFF phenomenon in postural control, implying that better stabilization and the benefit from larger postural sway may not coexist. Further studies are needed to clarify the suspensory strategy effects during perturbation and explain whether the suspensory strategy of knee flexion can lead to better stabilization in multiple environments.

### 4.2. Sensory integration

One of the aims of this study was to examine the effects of suspensory strategy of bending the knee on sensory integration. Time-frequency analysis of the COM or COP is a frequently employed method to explore sensory input and measure sensory reweighting (10, 15, 16, 31). In terms of energy content, prior studies have suggested that an increase in sensory energy within the 0–6.25 Hz frequency range correlates with an increase in the balance-related sensory input (10, 13). This study findings indicated that balance-related sensory input showed an increase during knee flexion in both AP and ML directions. These findings may further explain the fact that older people prefer to choose the suspensory strategy of knee flexion with a lowered COM for balance control when performing internal perturbations (4) as balance-related sensory functions diminish with age (7, 20, 39), and the increased sensory input from knee flexion may compensate for this sensory deficit. According to the descriptions of the balance-related sensory reweights in each frequency band from previous studies (10, 15, 31), in terms of the sensory weights in the AP direction, the results of the present study only showed a decrease in the vestibular sensory-related weights when the knee flexion was 65 °. Interestingly, in the ML direction, the present study showed that in the 65° knee flexion, the visual-related weights decreased, but the vestibular-related sensory weights increased (10, 15, 31). This suggests that the suspensory strategy by knee flexion increases the balance-related sensory input simultaneous with the changes in the balance-related sensory weights, and that this change is inconsistent in the AP and ML directions of balance. Therefore, a suspensory strategy of knee flexion may compensate for some deficits in the balance-related sensory acquisition to increase postural control and alter the balance-related sensory weights in the standing balance.

### 4.3. Regularity of postural sway

Typically, a decrease in sample entropy increases the regularity of postural sway, reflecting a shift toward more attention and less automatic control required to control balance. However, an increase in sample entropy decreases the regularity of postural sway, which in turn corresponds to less attention and more automatic control required for balance (11, 17, 19). According to this interpretation of sample entropy, the findings of this study support the fact that in the static standing position, knee flexion decreases the passive stability and increases the active stability, thereby requiring muscle activity and increased attention to postural stability (22, 27, 40). This phenomenon was only observed in the ML and vertical directions. This may not be a good indication of healthier balance control because most conceptualizations of static balance suggest that better static balance corresponds to more automatic control and less attentional involvement (20, 41). However, in daily life, it is not only important to automatically maintain static balance but also cope with unexpected perturbations. More attention to postural control may allow for quicker balance response and recovery when subjected to external perturbations. Moreover, some studies suggest that directing additional attention to balance might offer a more advantageous strategy, particularly for aging individuals or those grappling with compromised balance-related capacities (39). Additionally, this may aid in elucidating the findings of our previous study which showed that older adults are more inclined to adopt a suspensory strategy for postural sway (4). Declines in balance-related sensory functions and central motor abilities are common with age (12, 42). Consequently, it may be rational to intensify attentional allocation to maintain balance. Thus, adopting a suspensory strategy with knee flexion could serve as an effective means of directing more attention toward balance in the elderly. This study elucidated the nuanced relationship between knee flexion, postural sway regularity, and attentional demand.

### 4.4. Limitations

This study has several limitations. First, the study sample consisted of only young individuals, limiting the generalizability of the findings to other older age groups. Future research should include participants across a wider age range to examine the potential age-related differences in balance control during squatting. Second, this study focused solely on sensory input and sample entropy under open-eye conditions. Future investigations could consider incorporating other sensory conditions, such as eyes closed or altered visual feedback, to provide a more comprehensive understanding of the sensory contributions to balance control during squatting. This study examined three specific knee flexion angles (0°, 15°, and 65°) but did not include intermediate angles between 15° and 65°. Investigating a broader range of knee flexion angles would provide a more detailed understanding of how different angles affect balance control and the associated sensory input. Finally, this study did not collect electromyograms, and a more detailed analysis of the conclusions or muscle contributions may require support from these data. Addressing these limitations in future studies will enhance our knowledge of balance control during squatting and provide a more comprehensive understanding of the underlying mechanisms.

In conclusion, this study revealed that suspensory strategy by knee flexion led to an increased COM amplitude in healthy adults, and the suspensory strategy may be able to increase the functionality and attentional control of postural sway to some extent. Balance gains from suspensory strategy may be more appropriate for older adults with declining balance-related sensory, central processing, and musculoskeletal system function. However, it should be noted that this study population did not include older adults and more complex dynamic balances. Further research should be conducted to fully analyze the suspensory strategy. From a clinical perspective, this study provides new insights into a rehabilitative approach to balance control in older adults using a suspensory strategy. This work could also offer pertinent insights for the design of knee joint exoskeletons, particularly concerning the interplay between the use of knee joint exoskeletons, overall postural sway control, the potential effects of exoskeleton movement on the integration of sensory functions, and the attention of the user toward postural control.

## Data availability statement

The raw data supporting the conclusions of this article will be made available by the authors, without undue reservation.

## Ethics statement

The studies involving humans were approved by Faculty of Health Sciences at Hokkaido University. The studies were conducted in accordance with the local legislation and institutional requirements. The participants provided their written informed consent to participate in this study.

## Author contributions

LJ: Conceptualization, Data curation, Formal analysis, Investigation, Methodology, Software, Visualization, Writing – original draft, Writing – review & editing, Funding acquisition. SK: Conceptualization, Data curation, Investigation, Methodology, Project administration, Supervision, Writing – original draft, Writing – review & editing, Funding acquisition. TI: Conceptualization, Methodology, Project administration, Supervision, Writing – review & editing. YK: Conceptualization, Methodology, Supervision, Writing – review & editing. AC: Data curation, Supervision, Validation, Writing – review & editing. KY: Data curation, Supervision, Writing – review & editing. YW: Supervision, Writing – review & editing. MS: Conceptualization, Investigation, Methodology, Supervision, Writing – review & editing. HT: Conceptualization, Investigation, Methodology, Supervision, Writing – review & editing.

## Abbreviations

ANOVA: Analysis of variance (ANOVA)
AP: Anterior–posterior
BOS: Base of support
COM: Center of mass
COP: Center of pressure
ML: Medial-lateral
